# Analysis of Polymorphisms in the Mediator Complex Subunit 13-like (*Med13L*) Gene in the Context of Immune Function and Development of Experimental Arthritis

**DOI:** 10.1007/s00005-018-0516-8

**Published:** 2018-06-27

**Authors:** Samra Sardar, Katrine Kanne, Åsa Andersson

**Affiliations:** 1 Section for Molecular and Cellular Pharmacology, Department of Drug Design and Pharmacology, University of Copenhagen, Copenhagen, Denmark; 2 Present Address: Nordic Bioscience A/S, Copenhagen, Denmark; 3 Present Address: Novartis International AG, Copenhagen, Denmark; 4 Rydberg Laboratory of Applied Sciences, School of Business, Engineering and Science, Halmstad University, Halmstad, Sweden

**Keywords:** MED13L, THRAP2, Mediator complex, Collagen-induced arthritis, Rheumatoid arthritis, Congenic mice

## Abstract

The Mediator complex subunit 13-like (MED13L) protein is part of the multi-protein mediator complex and plays an important role in gene transcription. Polymorphisms in the *MED13L* gene have been linked to congenital heart anomalies and intellectual disabilities. Despite recent evidence of indirect links of MED13L to cytokine release and inflammation, impact of genetic variations in *MED13L* on immune cells remains unexplored. The B10.RIII and RIIIS/J mouse strains vary in susceptibility to induced experimental autoimmune disease models. From sequencing data of the two mouse strains, we identified six polymorphisms in the coding regions of *Med13L*. Using congenic mice, we studied the effect of these polymorphisms on immune cell development and function along with susceptibility to collagen-induced arthritis, an animal model for rheumatoid arthritis. Combining in vivo disease data, in vitro functional data, and computational analysis of the reported non-synonymous polymorphisms, we report that genetic polymorphisms in *Med13L* do not affect the immune phenotype in these mice and are predicted to be non-disease associated.

**Electronic supplementary material:**

The online version of this article (10.1007/s00005-018-0516-8) contains supplementary material, which is available to authorized users.

## Introduction

The Mediator complex subunit 13-like (MED13L) protein, also known as thyroid hormone receptor associated protein 2 (THRAP2) and Trap240-like (TRAP240L), is a recently identified molecule in humans (Musante et al. 2004). MED13L is a paralog of the closely related protein called Mediator complex subunit 13 (MED13), and is believed to have arisen from gene duplication events (Daniels et al. 2013). MED13L is one of the subunits of the dissociable CDK8 kinase module (CKM) of the Mediator, a multi-protein complex that plays an essential role during RNA polymerase II (pol II) gene transcription (Sato et al. 2004; Tsai et al. 2014). The CKM, consisting of the CDK8, CYCLIN C, MED12/MED12L, and MED13/MED13L subunits, acts as a molecular switch that regulates the Mediator co-activator function. The CKM associates with the Mediator to repress basal transcription of pol II-dependent genes and its dissociation from the Mediator has a stimulatory effect on transcription (Tsai et al. 2013). The MED13/MED13L subunit is critical for the CKM-dependent repression, as the strongest Mediator-CKM interaction involves a discrete contact through this subunit, thereby interfering with pol II recruitment and the holoenzyme formation (Knuesel et al. 2009; Tsai et al. 2013).

Mutations in the *MED13L* gene have established causation of transposition of the great arteries, a congenital heart anomaly (Adegbola et al. 2015; Lei et al. 2014). More recently, a wider spectrum of diseases has been linked to genetic variations in this gene and are collectively termed as *MED13L* haploinsufficiency syndrome, which is characterized by cardiac anomalies, developmental delay, distinctive facial features, intellectual disability, and in some cases craniosynostosis (Adegbola et al. 2015; Yamamoto et al. 2017). However, presentation of the *MED13L* haploinsufficiency syndrome varies among affected individuals, and not all patients have the associated cardiac phenotype (van Haelst et al. 2015). *MED13L* haploinsufficeinecy syndrome is a rare disease with only few cases reported worldwide (NIH rare diseases information database: https://rarediseases.info.nih.gov/diseases/12999/med13l-syndrome#ref_11391). Therefore, the complete spectrum of diseases associated with the syndrome and functional significance of *MED13L* variants is not completely elucidated.

To date, 22 disease-associated mutations have been reported in the *MED13L* gene, but the molecular pathways affected by these mutations, leading to the reported clinical phenotypes, are largely unknown (Musante et al. 2004; Yamamoto et al. 2017). *MED13L* has been reported to be involved in the Wnt, fibroblast growth factor (FGF), and retinoblastoma (Rb)/E2F pathways (Angus and Nevins 2012; Asadollahi et al. 2017; Utami et al. 2014). The Wnt pathway is an evolutionarily conserved signaling pathway that controls proliferation and differentiation of progenitor cells and is critical for several biological processes. Emerging studies have highlighted not only developmental but also a regulatory role for the Wnt signaling pathway in the immune system (Clevers and Nusse 2012; Staal et al. 2008; van Amerongen and Nusse 2009). Rheumatoid arthritis (RA) is a chronic autoimmune disease characterized by inflammation in joints, followed by destruction of cartilage and bone. In studies of inflamed RA joint synovial tissue, it was shown that high amounts of Wnt isoforms were expressed (Sen et al. 2000). Similar findings were found in a mouse model for RA, collagen-induced arthritis (CIA), where Wnt5a was highly expressed in synovial tissue of arthritic mice (Maeda et al. 2012). Signaling through the FGR receptors 1–4 is another biological pathway where a role for MED13L is implicated. FGF has an important role in immunity, in particular through the neutrophil chemotactic function exerted by FGF1 and FGF2 (Haddad et al. 2011).

Although the Rb-E2F pathway is well known to have a crucial role in physiological proliferation and oncogenesis, its role in immune modulation is more recently appreciated. Rb impacts development of the immune system by regulating various transcriptional pathways during progenitor differentiation, primarily common myeloid progenitors (Hutcheson et al. 2015).

Recent reports have also shown an indirect link of MED13L to inflammation, through activating transcription factor 4 (ATF4). Tomppo et al. (2012) reported that *MED13L* is acting downstream of ATF4 that regulates its expression levels, thereby pointing towards potential involvement of MED13L in pathways regulated by ATF4. ATF4 is a member of the activating transcription factor family and is essential in many biological mechanisms, such as in the stress response, medullary hematopoiesis and bone resorption (Ameri and Harris 2008). Several studies show that ATF4 participates in inflammatory responses and positively regulates the secretion of pro-inflammatory cytokines such as interleukin (IL)-6, IL-8 and interferon γ (Iwasaki et al. 2014; Sasaki et al. 2017; Zhang et al. 2013). Furthermore, a genome-wide association study (GWAS) suggested that a region in the proximity of the *MED13L* gene is associated with type 1 diabetes (Wellcome Trust Case Control Consortium 2007). Despite these reports on a potential role of *MED13L* in immune function and inflammation, the effect of genetic variations in this gene has not been studied in immune cells and animal models of autoimmune diseases.

Experimental animal models of RA are critical tools in furthering our understanding of disease pathogenesis, discovery of disease biomarkers, and in the development and testing of new therapies (Sardar and Andersson 2016). Among the available models, CIA is the most commonly used model. Based on its clinical and pathological similarities to RA, in addition to its reproducibility, the CIA model has been used extensively to identify potential disease pathways, including the role of individual cell types and inflammatory mediators in disease onset and progression. CIA can be induced in susceptible strains of rodents (mouse and rat) (Holmdahl et al. 2002).

Congenic mice are inbred strains of mice where a specific genetic region from one mouse strain has been introduced to a genetically different mouse strain by breeding. This breeding strategy, including selection- and background genotyping of each generation, makes it possible to study the role of naturally occurring polymorphisms for a particular phenotype and occupies a pivotal position in the elucidation of mechanisms in complex immune diseases such as RA (Rogner and Avner 2003).

In this study, we explored the effect of coding polymorphisms between two natural variants of the *Med13L* gene, found in the B10.RIII and RIIIS/J mouse strains that differ in their susceptibility to CIA and experimental autoimmune encephalomyelitis (EAE), a model for multiple sclerosis. We used BR.RIIIS/J-*Eae39r1* congenic mice that express the *Med13L* gene from the CIA and EAE resistant RIIIS/J donor strain on the genetic background of the B10.RIII, which is susceptible to the experimental autoimmune disease models. We found that *Med13L* polymorphisms between the B10.RIII and RIIIS/J mouse strains do not affect immune phenotype, CIA development and autoantibody production in these mice.

## Materials and Methods

### Mice

BR.RIIIS/J-*Eae39r* congenic mice were produced by introduction of the *Eae39r* fragment from the CIA resistant RIIIS/J donor strain, purchased from the Jackson Laboratory (Bar Harbor, ME, USA), to the CIA susceptible B10.RIII background strain, provided by J. Klein (Tübingen, Germany), as previously described (Lindvall et al. 2009). The sub-congenic line, BR.RIIIS/J-*Eae39r1*, was produced by further inter-crossing heterozygous BR.RIIIS/J-*Eae39r* mice. All mice were kept and bred, under standard conditions, in the animal facility at the Department of Drug Design and Pharmacology, Faculty of Health and Medical Sciences, University of Copenhagen, Denmark. Danish Animal Experiment Inspectorate license numbers: 2010/561-1920 and 2015-15-0201-00794.

### DNA Purification, Genotyping and Sequencing

Genomic DNA (gDNA) was purified from mouse ear or tail biopsies with a High Pure PCR template preparation kit (11796828001; Roche Holding AG, Basel, Switzerland) according to the manufacturer’s recommendation. Purified gDNA was used for genotyping by high resolution melting (HRM) single nucleotide polymorphism (SNP) genotyping for rs33583463 (5:118,596,773 bp, mouse genome assembly GRCm38) and by PCR-agarose gel electrophoresis method for homemade microsatellite marker D5tbxhm17 (5:119,660,373 bp, mouse genome assembly GRCm38). HRM analysis was carried out on Roche LightCycler 480 using High Resolution Melting Master (04909631001; Roche Holding AG, Basel, Switzerland) as described elsewhere (Thomsen et al. 2012). Microsatellite marker genotyping was determined by analyzing PCR products on a MegaBACE1000 genotyping system (Amersham Biosciences, Little Chalfont, UK), as previously described (Karlsson et al. 2003), or on a 3% agarose gel.

For DNA sequencing, all coding exons (and surrounding regions) of the *Med13L* gene for BR.RIIIS/J-*Eae39r1* congenic and B10.RIII control mice were amplified with specific primer pairs and sequenced by Sanger technology (GATC biotech, Germany). Primer sequences used for genotyping and sequencing are available upon request.

### Bioinformatics

The identified SNPs between B10.RIII and BR.RIIIS/J-*Eae39r1* mice were coding synonymous, not changing the amino acid and non-synonymous, if the amino acid was altered. The possible effects of amino acid alteration by non-synonymous variations were predicted by a web-based tool, sorting intolerant from tolerant (SIFT: http://siftdna.org/www/SIFT_dbSNP.html). This tool identifies the impact of amino acid substitution on protein function and phenotype alterations based on the type of amino acid change and evolutionary conservation of the position at which the change occurred, thereby distinguishing mutations more likely to be involved in disease (deleterious) from neutral (tolerated) polymorphisms (Ng and Henikoff 2003). It assigns scores to each residue, ranging 0–1, assuming that the most frequent amino acid across species is being better tolerated. The SNPs with score between 0.0 and 0.05 are considered deleterious, and variants with score between 0.05 and 1.0 are predicted to be tolerated. Variants with scores very close to 1.0 are more confidently predicted to be tolerated.

The orthologs of *Med13L* in closely related species were identified using the Ensembl database and evolutionary conservation of the regions around the amino acid changes in the protein was determined by multiple sequence alignment (MSA) by NCBI T-coffee server (http://www.ebi.ac.uk/Tools/msa/tcoffee/) (Di Tommaso et al. 2011). The results were imported into Jalview (Waterhouse et al. 2009) and SNP locations were marked.

### Induction and Evaluation of Collagen-Induced Arthritis

CIA was induced in 20 male mice, 8–10 weeks old, by intra-dermal injection of 100 µg bovine collagen type II (CII-7806; Sigma-Aldrich, St Louis, MO, USA) emulsified in incomplete Freund’s adjuvant (IFA) (F5506; Sigma-Aldrich, St Louis, MO, USA) at the base of the tail (day 0) followed by a booster dose of 50 µg bovine CII emulsified in IFA on day 35. Clinical disease was monitored in a blinded manner three times a week according to a scoring system whereby each inflamed toe (first phalanx excluded), all inflamed knuckles and inflamed wrist or ankle were assigned one point giving a maximum of 6 points per paw and a theoretical maximum score of 24 per mouse. According to the Danish Animal Experiment Inspectorate approved humane endpoints, mice receiving scores above 10 were euthanized.

### Enzyme-Linked Immunosorbent Assay for Anti-collagen Antibody Titers

For anti-collagen type II enzyme-linked immunosorbent assay (ELISA), sera were prepared from blood collected on day 0 and 15 (by submandibular bleeding), and on the last day of CIA (by cardiac puncture). The levels of CII-specific IgM, IgG_1_, IgG_2c_ and IgG_3_ antibodies were determined as previously described (Lindvall et al. 2009). Briefly, serum dilutions were applied to 96-well micro-titer plates (163320; Nunc maxisorp, Roskilde, Denmark) coated overnight with CII in phosphate buffered saline (PBS) (0.5 µg/well) and subsequently blocked with 1% bovine serum albumin (BSA)/PBS solution. The antigen–antibody binding signal was revealed by biotinylated secondary antibodies: goat anti-mouse IgM (1020-08), IgG_1_ (1070-08), IgG_2c_ (1079-08), and IgG_3_ (1100-08) (SouthernBiotech, Birmingham, AL, USA) and horseradish peroxidase (HRP)-conjugated avidin (554058, BD Pharmingen, San Jose, CA, USA) followed by detection with ABTS (2,2′-Azinobis [3-ethylbenzothiazoline-6-sulfonic acid]-diammonium salt) substrate (A1888; Sigma-Aldrich, St Louis, MO, USA). A SpectraMax Microplate Reader (Molecular Devices Corporation, Sunnyvale, CA, USA) was used to read the absorbance at a wavelength of 405 nm with a wavelength correction set to 492 nm. Pooled sera from arthritic mice were used as a standard and the antibody levels were measured as arbitrary concentrations.

### Preparation of Single-Cell Suspension

Spleen and thymus were harvested, and single-cell suspensions were prepared by mashing the tissue through a 40 µm cell strainer (431750; Corning, New York, USA). The spleen samples were treated with BD Lysing Buffer (555899; BD Biosciences, San Jose, CA, USA) to obtain erythrocyte-free cell suspensions for cell cultures and flow cytometry. Single-cell suspensions of thymus were only used for flow cytometric analysis.

### In Vitro Proliferation Assay of Splenic B and T Lymphocytes

Unsorted splenic lymphocytes (2 × 10^5^ cells per well) were incubated in complete Dulbecco’s modified eagle’s medium consisting of DMEM GlutaMAX-I, 5% fetal bovine serum, 1 mM Hepes, 50 µM 2-Mercaptoethanol, and 1% Penicillin/Streptomycin (Invitrogen, Thermo Fisher Scientific, Waltham, MA, USA), and stimulated for 48 h with titrated amounts of lipopolysaccharide (LPS) (L2880; Sigma-Aldrich, St Louis, MO, USA) or goat anti-mouse IgM F(ab′)_2_ (115-006-075; Jackson ImmunoResearch Laboratories, Inc. Baltimore, PA, USA), for B lymphocyte proliferation assay. For measuring the T cell proliferative response, 2 × 10^5^ splenic lymphocytes were stimulated for 48 h with titrated amounts of Concanavalin A (ConA), or a combination of purified anti-mouse CD3 antibody, clone 145-2C11 (550275; BD Biosciences, San Jose, CA, USA) and anti-mouse CD28 antibody, clone 37.51 (16-0281; eBioscience, San Diego, CA, USA). The cells were pulsed with 1 µCi per well ^3^H-thymidine (Perkin Elmer, Waltham, MA, USA) for the final 16–18 h, and incorporation was counted on a TopCount Scintillation Counter (Perkin Elmer, Waltham, MA, USA). All data are shown as mean counts per minute, indicating ^3^H-thymidine incorporation, in triplicate cultures.

### Measurement of IL-2 in Culture Supernatants

The amounts of murine IL-2 in supernatant from splenocyte cultures, stimulated with ConA or a combination of anti-CD3/anti-CD28 antibodies for 48 h, were determined by ELISA according to the manufacturer’s instructions (BD Pharmingen, San Diego, CA, USA). Briefly, 96-well micro-titer plates were coated overnight with anti-mouse IL-2 capture antibody, clone JES6-1A12 (554424, BD Pharmingen, San Diego, CA, USA) in PBS (0.1 µg/well) and subsequently blocked with 1% BSA/PBS solution for 2 h. Then 100 µl of the culture supernatants, and doubling dilutions (0–7000 pg/ml) of the mouse IL-2 standard (550069, BD Pharmingen, San Diego, CA, USA) were added to the wells and incubated overnight. 0.05 µg/well of biotinylated IL-2 detection antibody clone JES6-5H4 (554424, BD Pharmingen, San Diego, CA, USA) for 1 h, followed by 1:1000 dilution of Avidin-HRP for 45 min at room temperature, and finally ABTS substrate were each applied to the wells. Absorbance was read at a wavelength of 405/nm and was converted to IL-2 concentration (pg/ml) using a linear standard curve.

### Flow Cytometric Analysis

Single-cell suspensions of spleen and thymus tissue were prepared in complete DMEM medium as described above. Splenic lymphocytes were stained with anti-mouse CD4-phycoerytherin (CD4-PE), anti-mouse CD8-phycoerytherin-cyanine 5 (CD8-PE-Cy5), and anti-mouse CD19 fluorescein isothiocyanate antibodies, all purchased from BD Biosciences (San Jose, CA, USA) (Supplementary Fig. 1). Thymocytes were only stained with anti-mouse CD4-PE and anti-mouse CD8-PE-Cy5 antibodies to study various sub-classes of T lymphocytes (Supplementary Fig. 2). 1 × 10^6^ cells were pre-incubated with 1 µg of Fc receptor block (anti-CD16/CD32 antibody clone 93; 14-0161-81, eBioscience, San Diego, CA, USA), prior to staining with 0.1 µg of respective antibodies for 15 min on ice. The stained cells were analyzed using Gallios Flow Cytometer (Beckman Coulter, Brea, CA, USA) and FlowJo software (Tree Star Inc, Ashland, OR, USA).

### Statistical Analysis

Statistics were calculated in GraphPad Prism version 7.03. All CIA (except disease incidence and ethical survival data), ELISA, in vitro proliferation and flow cytometry data were calculated with Mann–Whitney *U* test. For CIA incidence and ethical survival data, Chi-square test and Fischer’s exact test were applied, respectively. *p* values less than 0.05 were considered significant in all cases.

## Results

### Coding Polymorphisms in *Med13L*

**Fig. 1:**
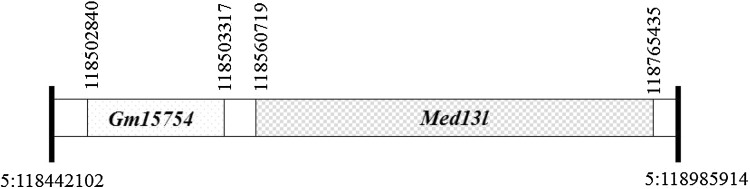
Genetic elements located in the *Eae39r1* congenic fragment on mouse chromosome 5. The region spans over 0.5 mega base pairs, whereby rs33731225 (5:118,442,102 bp) and rs6255362 (5:118,985,914 bp), shown as thick lines, mark the boundaries of the fragment. The fragment consists of a pseudogene *Gm15754* (5:118,502,840–118,503,317) and a protein coding gene *Med13L* (5:118,560,719–118,765,435). Coordinates are based on Ensembl release 74 (mouse genome assembly GRCm38). The picture is a graphic depiction and not to scale

The BR.RIIIS/J-*Eae39r1* strain is genetically similar to B10.RIII except for the locus *Eae39r1*, which was introduced from the RIIIS/J mouse strain. *Eae39r1* comprises a small region with only two genetic elements, *Gm15754* and *Med13l* (Fig. 1). *Gm15754* is a pseudogene, defined as genomic remnants of ancient protein-coding genes, and data on the functionality is controversial (Li et al. 2013; Tutar 2012). *Med13L* is a protein-coding gene with 96% similarity to human *MED13L*.

Sequencing of coding regions of the *Med13L* gene from B10.RIII and BR.RIIIS/J-*Eae39r1* mice, revealed six coding SNPs (Table 1). Two out of the six identified variants were coding non-synonymous, thereby leading to an alteration of the amino acid sequence of the protein product, while the rest were synonymous SNPs.

**Table 1:** List of SNPs in the coding region of *Med13L* in the B10.RIII and BR.RIIIS/J-*Eae39r1* strains of mice

Variation ID	Location^a^	Exon^b^	Codon^c^	Functional class^d^	Amino acid change^e^	Residue
rs13478486	5:118,731,440	13	GA**A**/GA**C**	CNSyn	E/D	814
rs38736382	5:118,742,369	17	CT**C**/CT**T**	CSyn	L	1175
rs33215085	5:118,742,508	17	**G**TA/**A**TA	CNSyn	V/I	1222
rs33658171	5:118,742,588	17	GC**C**/GC**T**	CSyn	A	1248
rs36568864	5:118,745,106	18	GG**C**/GG**T**	CSyn	G	1343
rs33709896	5:118,748,751	21	CC**C**/CC**T**	CSyn	P	1572

^a^Coordinates according to Ensembl release 74 (mouse genome assembly GRCm38)

^b^Exon numbers are relative to the Med13l-201 transcript (length: 9296 bps, coding exons: 31, translation length: 2207 residues)

^c^Written as B10.RIII/ BR.RIIIS/J-*Eae39r1*; the altered nucleotides are shown in bold

^d^Functional class, *CNSyn* coding non-synonymous SNP, *CSyn* coding synonymous SNP

^e^E-glutamate, d-aspartate, l-leucine, V-valine, I-isoleucine, A-alanine, G-glycine, P-proline

Comparison of the six SNPs in a number of laboratory mouse strains showed that the B10.RIII and RIIIS/J alleles are found in other mouse strains as well. The variations found in B10.RIII, shared with for example the C57Bl/6 and NOD mouse strains, are, however, less frequent (Table 2).

**Table 2:** Allelic variation in the coding region of *Med13L*

Mouse strain^a^	rs1347848^b^ MAF: 0.22^c^	rs38736382 MAF: 0.39	rs33215085 MAF: 0.33	rs33658171 MAF: 0.39	rs36568864 MAF: 0.39	rs33709896 MAF: 0.39
B10.RIII	A/A	C/C	G/G	C/C	C/C	C/C
RIIIS/J	C/C	T/T	A/A	T/T	T/T	T/T
129S1/SvImJ	C/C	T/T	A/A	T/T	T/T	T/T
A/J	C/C	T/T	A/A	T/T	T/T	T/T
BALB/cJ	C/C	T/T	A/A	T/T	T/T	T/T
C3H/HeJ	C/C	T/T	A/A	T/T	T/T	T/T
C57BL/6NJ	A/A	C/C	G/G	C/C	C/C	C/C
DBA/2J	C/C	T/T	A/A	T/T	T/T	T/T
NOD/ShiLtJ	A/A	C/C	G/G	C/C	C/C	C/C

^a^Seven common laboratory mouse strains were compared to the B10.RIII and RIIIS/J parental strains (http://www.ensemble.org)

^b^Variant ID

^c^Minor allele frequencies (MAF) are based on data from the Mouse Genome Project (http://www.sanger.ac.uk/science/data/mouse-genomes-project)

### Analysis of *Med13L* Polymorphisms

**Fig. 2:**
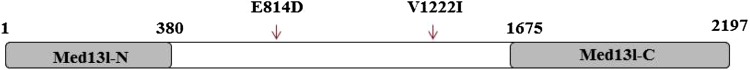
Graphical presentation of the Med13L protein and amino acid-altering SNPs between B10.RIII and BR.RIIIS/J-*Eae39r1* mice. The conserved N terminal (Med13l_N) and C terminal (Med13l_C) domains are shown in grey. Numbers representing amino acid residue and location of amino acid changes due to coding non-synonymous SNPs between B10.RIII and BR.RIIIS/J-*Eae39r1* mice (E814D and V1222I) are shown by arrows

Since rs13478486 and rs33215085 (Fig. 2) alter the amino acid sequence of the encoded protein, they were selected for further analysis. The SNP rs13478486 (E814D) changes glutamate (E) in B10.RIII to aspartate (D) in BR.RIIIS/J-*Eae39r1* mice. Both are hydrophilic (acidic) amino acids with similar structures, except for one extra methylene (–CH_2_) group in the side chain of glutamate that increases the molecular weight (mw) from 133 Dalton (Da) for aspartate to 147 Da for glutamate. This extra carbon atom in glutamate also changes the plane of symmetry for the amino acid to some extent, but its impact on the protein structure and function is context-dependent (Young and Ajami 2000).

The other SNP, rs33215085 (V1222I), replaces valine (V) in B10.RIII with isoleucine (I) in BR.RIIIS/J-*Eae39r1* mice. Both valine (mw: 117 Da) and isoleucine (mw: 131 Da) are non-polar hydrophobic amino acids that participate in hydrophobic interactions and determine the tertiary structure of the protein. Although their structure only varies by the presence of one extra methyl (–CH_3_) group in isoleucine, altered function (ligand selectivity) was reported for a protein where V was substituted by I (Yuan et al. 2010).

Taken together, the amino acid substitutions between B10.RIII and BR.RIIIS/J-*Eae39r1* mice, as a result of rs13478486 and rs33215085, do not change the chemical properties of the protein to a large extent, and may not affect the three-dimensional structure and stability of the protein.

### SIFT Prediction and Evolutionary Conservation of *Med13L* Mutation Sites

**Fig. 3:**
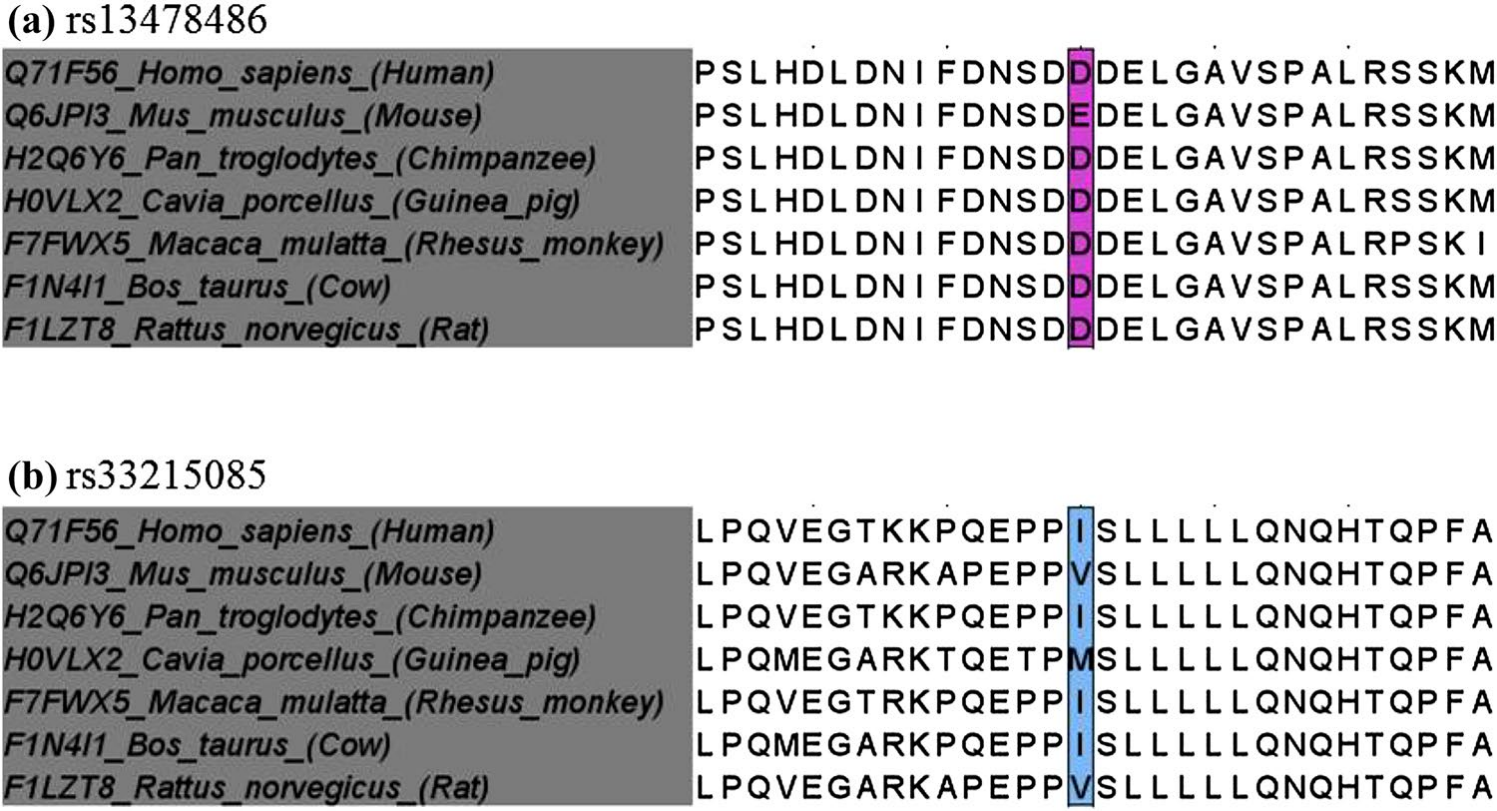
Protein sequence alignment across species performed by the T-coffee webtool. The regions flanking rs13478486-E814D (**a**) and rs33215085-V1222I (**b**) were aligned to Med13L orthologs in human, chimpanzee, guinea pig, monkey, cow and rat. The sequence identifier is written as Uniprot id of protein_Biological name of organism_common name of organism

It has previously been reported that disease-associated mutations are more likely to occur in functionally important regions of proteins that are evolutionary conserved (Mooney and Klein 2002; Mooney 2005). To investigate the evolutionary conservation of the regions with identified amino acid alterations in Med13L, MSA was performed with closely related species. We found that the region around E814D is more conserved than V1222I (Fig. 3), and that the nature of amino acid substitutions at these positions is not unique but observed naturally in some closely related orthologs. These findings indicate that amino acid variations caused by rs13478486 and rs33215085 are present in some other organisms and are less likely to be disease-related mutations. This goes in line with our SIFT predictions for rs13478486 (score 0.32) and rs33215085 (score 0.46) indicating that both variations are likely to be tolerable and non-disease causing.

### *Med13L* Polymorphisms Do Not Affect Development of CIA and Anti-CII Antibody Titers

**Fig. 4:**
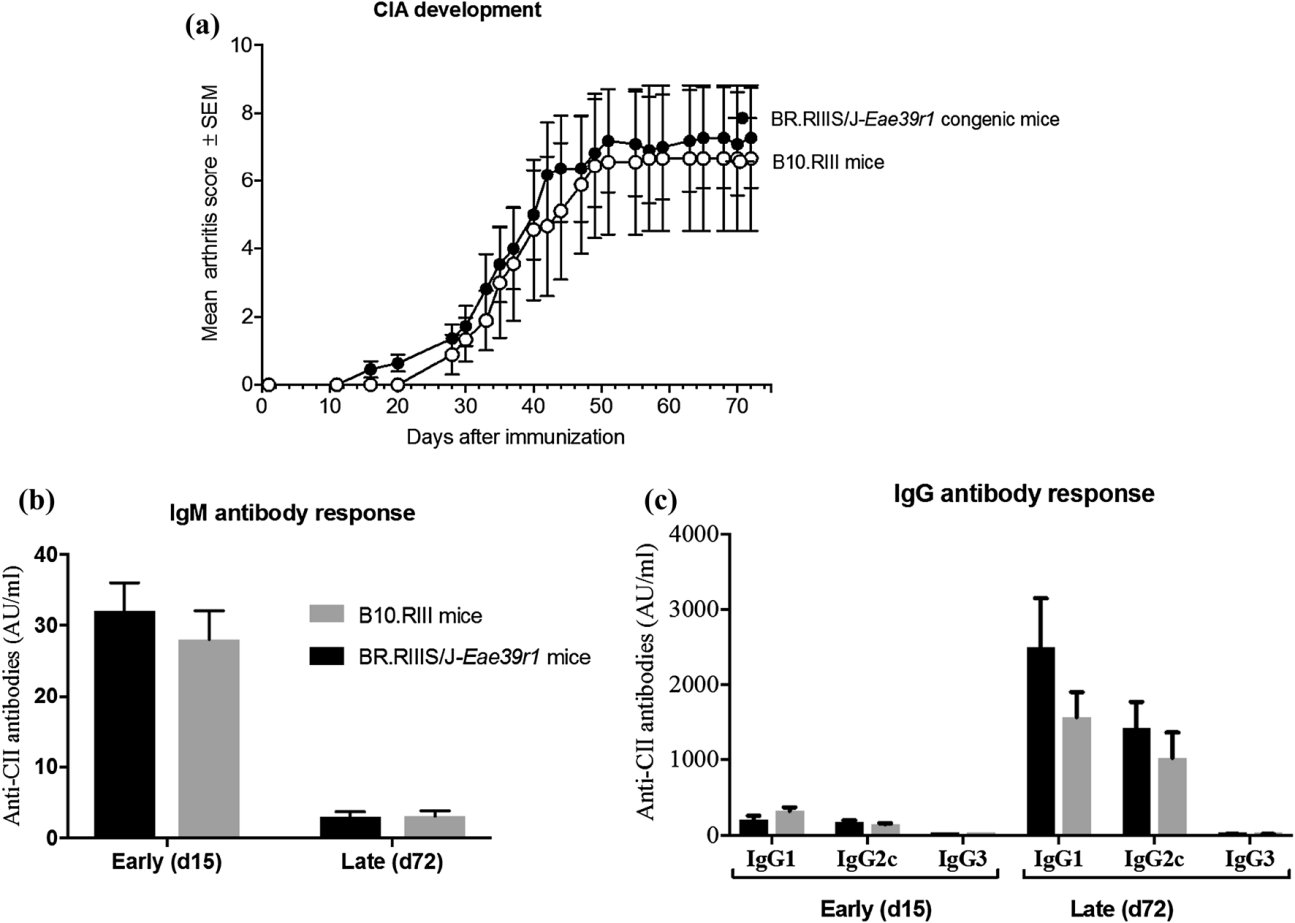
Collagen-induced arthritis (CIA) development and progression in B10.RIIIS/J*-Eae39r1* mice in comparison to B10.RIII littermate control mice, represented as mean arthritis score for each group ± standard error of the mean (SEM) (**a**); IgM (**b**) and IgG (**c**) anti-collagen type II antibody responses in early pre-clinical CIA, measured on day 15, and late clinical CIA, measured on day 72. (*n* = 9 for B10.RIIIS/J*-Eae39r1* and *n* = 11 for B10.RIII)

Considering the putative role of *Med13L* in autoimmunity, we studied the in vivo effect of the identified *Med13L* SNPs in the CIA mouse model. Upon induction of arthritis with CII/IFA, we found that both B10.RIII and BR.RIIIS/J-*Eae39r1* congenic mice developed progressive arthritis over the trial period and that there was no significant difference in disease onset, incidence, and severity between the groups (Fig. 4a; Table 3). Moreover, anti-CII antibody levels, measured during the pre-clinical phase (day 15) and late clinical phase (day 72), were comparable in both groups of mice (Fig. 4b, c). Taken together, both B10.RIII and BR.RIIIS/J-*Eae39r1* mice developed progressive CIA with corresponding rise in anti-CII antibodies of different isotypes, indicating that *Med13L* SNPs do not influence CIA susceptibility and autoantibody response in these mice upon immunization.

**Table 3:** Collagen-induced arthritis (CIA) diseases phenotypes in B10.RIII and BR.RIIIS/J-*Eae39r1* mice

Disease phenotype	BR.RIIIS/J-*Eae39r1* congenic (*n* = 9)	B10.RIII littermate controls (*n* = 11)	*p* value^a^
Incidence	5/9 (55.6%)	10/11 (90.9%)	0.413
Mean max score^b^	6.7 ± 2.2	7.4 ± 1.5	0.749
AUC^c^	99.1 ± 33.6	110.3 ± 24.7	0.599
Mean day of disease onset	30.2 ± 1.4	26.1 ± 3.5	0.582
Number of euthanized mice^d^	5/9 (55.6%)	6/11 (54.5%)	0.964

^a^Statistics were calculated using Chi-squared test for incidence and number of mice euthanized and Mann–Whitney *U* test for the other parameters

^b^Mean max score, indicating CIA severity, is the mean (± SEM) of the maximum score for all affected mice in the group

^c^Area under curve (AUC) is the mean (± SEM) of the sum of scores for mice of the corresponding genotype (day 28–72)

^d^Mice having a score above 10 were euthanized during the experiment as per recommendations of animal welfare authorities

### *Med13L* Polymorphisms Do Not Affect Lymphocyte Development and Function

We further sought to examine the effect of the *Med13L* SNPs in lymphoid cell lineage development and function that might be of importance for other immune-mediated disorders. Therefore, we characterized the lymphocyte populations in naïve B10.RIII and BR.RIIIS/J-*Eae39r1* mice by flow cytometry. The ratio between the splenic B- and T-cell fractions was found decreased in BR.RIIIS/J-*Eae39r1* mice (*p* = 0.0571). This could be due to a decreased relative number of B cells and a slightly increased proportion of T cells, but no significant difference in the proportion of B cells, T cells, CD4^+^ and CD8^+^ T cells in spleen was observed (Table 4). Furthermore, the proportion of thymocyte subpopulations was comparable between B10.RIII and BR.RIIIS/J-*Eae39r1* mice, suggesting that the *Med13L* polymorphisms have no large effect on B and T lymphocyte development.

**Fig. 5:**
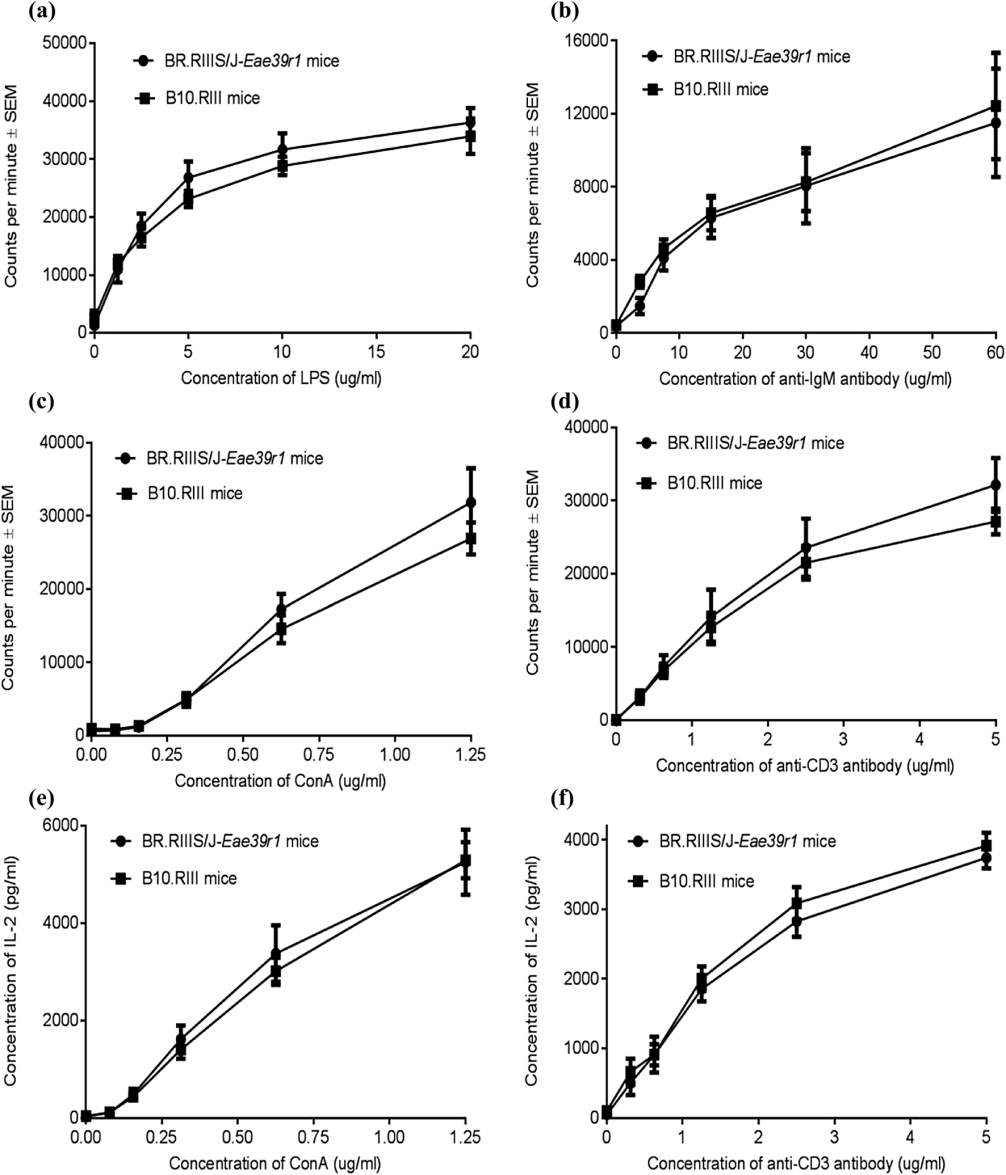
In vitro proliferative responses of splenic B and T lymphocytes from B10.RIII and BR.RIIIS/J-*Eae39r1* mice. ^3^H-thymidine incorporation, represented as counts per minute ± SEM, of B cells following in vitro stimulation with: (**a**) titrated concentrations (0–10 µg/ml) of LPS; (**b**) titrated concentrations (0–60 µg/ml) of anti-IgM antibody. ^3^H-thymidine incorporation of CD4^+^ T cells following in vitro stimulation with: (**c**) titrated concentrations (0–1.25 µg/ml) of ConA; (**d**) a combination of titrated concentrations (0–3 µg/ml) of anti-CD3 antibody and fixed concentration of anti-CD28 antibody (3 µg/ml). (**e, f**) IL-2 production (mean ± SEM) in the supernatant of T cells stimulated with ConA or anti-CD3/CD28 antibodies

To determine whether the *Med13L* polymorphisms would influence lymphocyte activation, the proliferative response of in vitro stimulated splenic lymphocytes from naïve B10.RIII and BR.RIIIS/J-*Eae39r1* mice was analyzed. No difference in proliferation of B cells upon stimulation with either LPS or anti-IgM was observed (Fig. 5a, b). Furthermore, no difference in proliferation or IL-2 production by T cells, following stimulation with ConA or anti-CD3/CD28 antibodies, was observed when comparing BR.RIIIS/J-*Eae39r1* mice with B10.RIII littermate controls (Fig. 5c–f). These data suggest that the *Med13L* polymorphisms between B10.RIII and BR.RIIIS/J-*Eae39r1* have no effect on numbers and activity of B- and T lymphocytes.

**Table 4:** Flow cytometry profiles of splenocytes and thymocytes from B10.RIII and BR.RIIIS/J-*Eae39r1* mice

	**Spleen**		
	B10.RIII	BR.RIIIS/J-*Eae39r1*	p-value^a^
CD19^+^ B cells (%)^b^	51.3 ± 2.4^c^	46 ± 1.4	0.200
T cells (%)	23.2 ± 3.3	29.2 ± 1.4	0.343
CD4^+^ T cells (%)	13.7 ± 1.9	17 ± 0.9	0.314
CD8^+^ T cells (%)	9.5 ± 1.4	12.2 ± 0.7	0.343
B/ T cell ratio	2.3 ± 0.3	1.6 ± 0.1	0.057

	**Thymus**		
	B10.RIII	BR.RIIIS/J-*Eae39r1*	p-value
CD4^+^ CD8^+^ T cells (%)	63.6 ± 4.5	57.1 ± 8.9	0.486
CD4^+^ CD8^-^ T cells (%)	3.9 ± 0.2	3.0 ± 0.4	0.171
CD4^-^ CD8^+^ T cells (%)	1.2 ± 0.1	1 ± 0.1	0.542
CD4^-^ CD8^-^ T cells (%)	28.6 ± 4.4	36.3 ± 9.6	0.486

^a^*p*-values were calculated with Mann-Whitney U test

^b^Data represent relative numbers (%) of B lymphocytes, T lymphocytes (measured as the sum of CD4^+^ and CD8^+^ T-cells), CD4^+^ and CD8^+^ T lymphocytes and ratio of B to T lymphocytes in spleen, and CD4^+^CD8^+^ double positive (DP), CD4^+^CD8^-^ and CD4^-^CD8^+^ single positive (SP) and CD4^-^CD8^-^ double negative (DN) thymocytes.

^c^Data are presented as mean ± SEM for each group where n=4

## Discussion

MED13L is an important component of the Mediator complex, responsible for bridging the RNA pol II enzyme and transcription factors to initiate transcription of protein-coding genes and non-coding RNA genes (Poss et al. 2013). *MED13L* is an evolutionarily conserved gene and the encoded protein share about 96% similarity with the mouse ortholog (Musante et al. 2004). Polymorphisms in this gene have been associated with cardiac and neurological phenotypes in humans (van Haelst et al. 2015; Yamamoto et al. 2017). However, the complete spectrum of diseases associated with *MED13L* polymorphisms and significance of these variations in relation to molecular pathways, are yet to be identified.

Based on reports that MED13L is involved in Wnt, FGF, Rb/E2F and ATF4 related pathways that play critical roles in lymphocyte development and function, in addition to inflammatory responses (Ameri and Harris 2008; Asadollahi et al. 2017; Clevers and Nusse 2012; Hutcheson et al. 2015; Kitajima and Takahashi 2017; Markey et al. 2007; Staal et al. 2008; Tomppo et al. 2012; van Amerongen and Nusse 2009; Zhang et al. 2013), we studied *Med13L* polymorphisms in the context of immune cell activation and pathogenesis of RA, a chronic inflammatory autoimmune disorder. From recent CIA studies, where mice with larger congenic fragments, including *Med13L*, developed significantly more severe arthritis (unpublished data), we wanted to investigate whether coding polymorphisms in *Med13L* might affect the disease phenotype, and/or development and function of lymphocytes involved in immune pathogenesis of the disease (Holmdahl et al. 2002; Sardar and Andersson 2016). Through extensive breeding and testing of congenic mice, BR.RIIIS/J-*Eae39r1* mice were established carrying only a 0.5 Mega base pair congenic fragment from the RIIIS/J strain, in which the only protein-coding gene is *Med13L* (Fig. 1). This makes these mice more valuable than knockout mice in terms of standardized genetic-background effects and ability to dissect out the effects of a particular allele(s) with natural variation (Rogner and Avner 2003).

MED13L has been shown to be involved in the Wnt and Rb/E2F signaling pathways (Angus and Nevins 2012; Utami et al. 2014) and the importance of Wnt signaling in T- and B-cell development in thymus and bone marrow, respectively, and their peripheral activation, is underpinned by a number of in vivo and in vitro studies. The thymi of mice deficient in Wnt (1 and 4) proteins show decreased number of mature CD4^+^ and CD8^+^ T cells (Mulroy et al. 2002), while activation of the Wnt pathway in transgenic mice, overexpressing activated forms of β-catenin, led to the generation of more thymocytes (Mulroy et al. 2003). Moreover, in vitro inhibition of Wnt proteins in fetal thymocyte cultures inhibits thymocyte differentiation (Staal et al. 2001). B-cell progenitors in bone marrow also express Wnt genes at various developmental stages and culturing mouse fetal pro-B cells in Wnt protein (3A)-conditioned medium leads to increased proliferation (Reya et al. 2000). Additionally, reduced numbers of CD19^+^ B lymphocytes in bone marrow and periphery have been observed with transgenic expression of a canonical Wnt inhibitor, kallistatin (McBride et al. 2017). In accordance with this, defects in T- and B-cell development in mice deficient for the Wnt-responsive transcription factors TCF1 and LEF1 are observed (Okamura et al. 1998; Reya et al. 2000). Furthermore, Markey et al. (2007) demonstrated that the deficiency of Rb protein in adult fibroblast cells is associated with down-regulation of genes involved in immune functions, with concomitant up-regulation of cell cycle genes thereby highlighting that Rb lies at the crossroads of proliferation and the immune response. It has been proposed that Rb directly regulates pro-inflammatory signaling and its inactivation has been associated with increased levels of pro-inflammatory cytokines, including chemokine (C-X-C motif) ligand 1 and 2 and IL-8 (Kitajima and Takahashi 2017). Thus, it is reasonable to expect that MED13L would be important for diseases within the immune system and for having modulating effects in the immune response.

We identified six SNPs in the coding region of *Med13L*, when comparing the genomic sequence of B10.RIII and BR.RIIIS/J-*Eae39r1* mice, and studied the SNPs putative functional effects on CIA development and immune cell activity, in addition to computational analysis. As synonymous SNPs are more likely to be neutral and tolerable in nature (Vitkup et al. 2003), our computational analysis and comparison of allelic variations in common laboratory mouse strains focused on the non-synonymous SNPs rs13478486 (E814D) and rs33215085 (V1222I). E814D corresponds to position 819 in the human protein and data on disease associated SNPs shows that it lies in a cluster with four known mutations S747Y, P835L, R842, D860G that are associated with mild to severe MED13L haploinsufficiency syndrome (Asadollahi et al. 2017). While the significance of these variants is uncertain, D860G is predicted to affect the secondary structure of MED13L. Patients with these mutations did not have any reported immune phenotypes, which goes in favor of our findings. However, these studies did not follow-up the patients over a long time so the possibility of later-onset immune mediated diseases cannot be excluded. V1222I corresponds to position 1223 in the human protein and a coding SNP rs748598145 (I1223M) has been reported for humans, but with no associated phenotype (NCBI SNP database: https://www.ncbi.nlm.nih.gov/SNP/).

Comparison between the two SNPs showed that V1222I has higher SIFT score and less conservation at the site of amino acid change than E814D, indicating that V1222I is more likely to be benign in nature, among the two variants under consideration. This finding is intriguing as V1222I lies at the minus 2-position of a LxxLL motif in both mouse and human protein. LxxLL motifs are present in many transcription factors and cofactors including components of the Mediator complex (Chen and Roeder 2011). These participate in many protein–protein interactions associated with different aspects of transcriptional regulation and have recently been implicated in various leukemias. The amino acids surrounding LxxLL motif are proposed to affect the binding affinity of these interactions and can alter the function of the protein (Plevin et al. 2005). On the contrary, we found that V1222I does not affect the function of Med13L in relation to immune phenotype and CIA development in BR.RIIIS/J-*Eae39r1* mice. This could be explained by chemical and structural similarities between valine and isoleucine, as reflected in the benign prediction of SIFT score for the amino acid switch.

MED13L has a putative link to RA pathogenesis through the Wnt and FGF pathways (Malemud 2007, 2013; Ye et al. 2015) and in a GWAS including RA patients and healthy controls from Korea, the homologous *Eae39r* region on human chromosome 12, including the *MED13L* gene, was weakly associated (Freudenberg et al. 2011).

Therefore, we used the classical CIA experimental model of RA to study the in vivo effects of *Med13L* polymorphisms. We have demonstrated that mice carrying reported *Med13L* SNPs develop CIA and mount an anti-collagen type II autoantibody response to the same extent as their non-mutant littermate controls upon induction with CII. Although a trend towards lower B to T lymphocyte ratio was observed in BR.RIIIS/J-*Eae39r1* mice, the difference was not significant and did not affect development of autoimmune arthritis in these mice. One emerging question is whether the polymorphisms found in the *Med13L* gene could have implications for disease development in other arthritis models like the KBxN serum-transfer arthritis model, the SKG model, and for CIA in the DBA/1 strain. The genetic background of KBxN is a cross between the C57Bl/6 and NOD mouse strains (Kouskoff et al. 1996), which both share the same alleles as B10.RIII in the *Eae39r1* locus (Table 2). The original SKG strain has the BALB/c genetic background (Sakaguchi et al. 2003), which share the RIIIS/J alleles in *Eae39r1*. Since we do not find any differences in arthritis susceptibility between mice with B10.RIII background and different alleles in the *Eae39r1* locus, we find it unlikely that the polymorphisms in *Med13L* would influence disease development in other arthritis susceptible strains. Concerning DBA/1, which is a commonly used mouse strain for CIA studies (Holmdahl et al. 1989; Trentham 1982), the map of SNPs in different databases is not as comprehensive as for other mouse strains. For one of the SNPs in *Med13L* (rs13478486), DBA/1 has the same allele as DBA/2 (http://www.informatics.jax.org/snp/ and Table 2), which makes it likely that DBA/1 carries the same alleles as DBA/2 in the whole locus and would be similar to RIIIS/J. Our conclusion from the results of our present investigation is that *Med13L* is not of crucial importance for disease development in CIA, but to make a final conclusion, the polymorphisms would need to be inferred in other arthritis susceptible strains and be tested for arthritis development in this context.

To summarize, we propose that the amino acid substitutions caused by rs13478486 (E814D) and rs33215085 (V1222I) variations in *Med13L*, do not affect arthritis development in the CIA model and lymphocyte development and function in general, and are less likely to be associated with immune-related diseases.

## Conclusion

In studies of *Eae39r1* congenic mice, we have shown that coding polymorphisms in the *Med13L* gene does not influence the function of the Med13L protein in terms of immune phenotype. We conclude that the investigated natural polymorphisms in *Med13L* do not play a critical role in autoimmune arthritis development, lymphocyte numbers or function. However, the role for Med13L in other inflammation- and autoimmunity-related pathways warrants further investigation.

## Electronic supplementary material

Below is the link to the electronic supplementary material.

**Supplementary Fig. 1** Flow cytometry with spleen cells. The antibody staining of cells is described in the Material section. Lymphocytes were gated in a Forward/Side Scatter dot plot (a). In the lymphocyte gate, B-cells were identified as CD19^+^ cells (FL1) and CD4^+^ T cells were gated in FL2 (b). CD8^+^ T cells were gated in FL4 (c). (TIF 1142 KB)

**Supplementary Fig. 2** Flow cytometry with thymocytes. The antibody staining of cells is described in the Material section. Thymocytes were gated in a Forward/Side Scatter dot plot (a). In the thymocyte gate, CD4^+^ T cells were gated in FL2 and CD8^+^ T cells in FL4 (b). The thymocyte population was divided into CD4^+^CD8^+^; CD4^+^CD8^+^; CD4^–^CD8^+^; and CD4^–^CD8^–^. (TIF 1142 KB)
