# Supplementary figures and images for: Analysis of Polymorphisms in the Mediator Complex Subunit 13-like (Med13L) Gene in the Context of Immune Function and Development of Experimental Arthritis
Source: Arch Immunol Ther Exp (Warsz). 2018 Jun 27;66(5):365–77. doi: 10.1007/s00005-018-0516-8 (PMC6154033; doi:10.1007/s00005-018-0516-8)

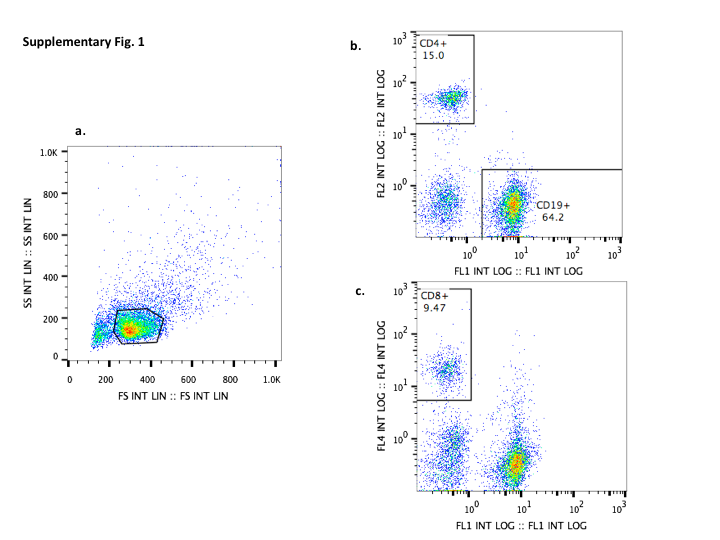

Supplement: Supplementary file 1 — Supplementary Fig. 1 Flow cytometry with spleen cells. The antibody staining of cells is described in the Material section. Lymphocytes were gated in a Forward/Side Scatter dot plot (a). In the lymphocyte gate, B-cells were identified as CD19+ cells (FL1) and CD4+ T cells were gated in FL2 (b). CD8+ T cells were gated in FL4 (c). (TIF 1142 KB) [file 5_2018_516_MOESM1_ESM.tif]

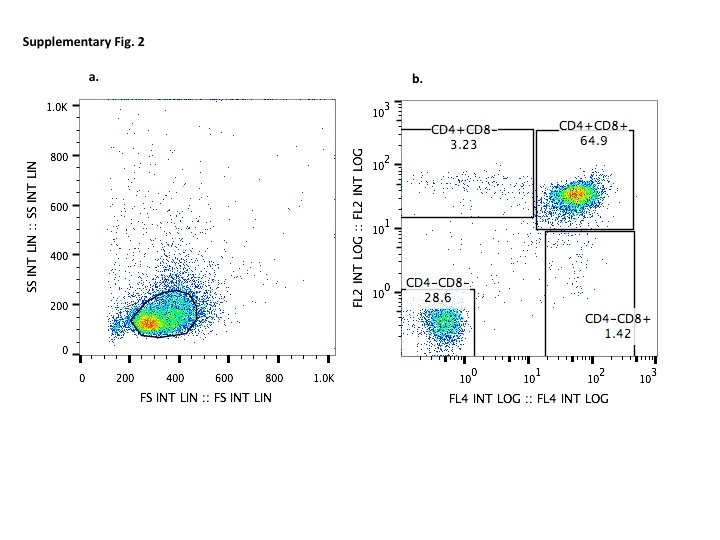

Supplement: Supplementary file 2 — Supplementary Fig. 2 Flow cytometry with thymocytes. The antibody staining of cells is described in the Material section. Thymocytes were gated in a Forward/Side Scatter dot plot (a). In the thymocyte gate, CD4+ T cells were gated in FL2 and CD8+ T cells in FL4 (b). The thymocyte population was divided into CD4+CD8+; CD4+CD8+; CD4–CD8+; and CD4–CD8–. (TIF 1142 KB) [file 5_2018_516_MOESM2_ESM.tif]
